# Investigating neutrophil cell death in TB pathogenesis

**DOI:** 10.12688/gatesopenres.13472.1

**Published:** 2021-12-07

**Authors:** Kimone L Fisher, Kerishka Rajkumar-Bhugeloo, Denelle Moodley, Thabo Mpotje, Duran Ramsuran, Thumbi Ndung'u, Mohlopheni J Marakalala

**Affiliations:** 1 Africa Health Research Institute, Durban, KwaZulu-Natal, 4001, South Africa; 2 School of Laboratory Medicine and Medical Sciences, University of KwaZulu Natal, Durban, KwaZulu-Natal, 4001, South Africa; 3 Division of Infection and Immunity, University College London, London, UK; 4 HIV Pathogenesis Programme, Doris Duke Medical Research Institute, University of KwaZulu Natal, Durban, KwaZulu-Natal, 4001, South Africa

**Keywords:** Neutrophils, Tuberculosis, NETosis

## Abstract

**Background: **Neutrophils are one of the major early role players in antimycobacterial immunity. Upon infection, neutrophils can undergo NETosis, a cell death characterized by release of neutrophil extracellular traps (NETs). The role of NETosis in TB progression remains poorly characterized. We aim to characterize mechanisms underlying NETosis during TB pathogenesis by identifying genes that drive the cell death, and to determine their potential as markers of disease progression in high-risk individuals. Finally, we intend to evaluate neutrophil associated genes as targets for host directed therapy to reduce pathological damage caused by NETosis. **Methods: **Quantitative PCR will be used to quantify expression of specific genes identified in the blood of individuals with active lung disease (n=30), compared to those from healthy (n=30) and latently infected individuals (LTBI) (n=30). In addition, temporal events associated with NETosis will be measured using live microscopy in a neutrophil in vitro model of *Mycobacterium tuberculosis *(Mtb) infection. Candidate genes found to be associated with NETosis will be targeted with pharmaceutical inhibitors. **Conclusion: **Genes associated with neutrophil mediated cell death may serve as potential biomarkers of pathological damage and disease progression, as well as targets for host-directed therapy.

## Background

Tuberculosis, caused by *Mycobacterium tuberculosis* (Mtb), is responsible for approximately 1.5 million deaths annually ^1^. Understanding lung immunity during TB progression is important for the identification of disease biomarkers. During initial TB infection, an immune structure known as a granuloma, forms to control and prevent the spread of Mtb ^2^. The granuloma is a highly organized immune structure composed of macrophages surrounded by a layer of epithelioid cells and multinucleated giant cells, with a lymphocytic cuff at the periphery ^3^. Neutrophils are also found in regions adjacent to caseum or necrotic regions of the granuloma ^4^. Understanding the contribution of neutrophils to lung associated tissue damage is important to determine mechanisms that lead to TB associated pathology. Neutrophils are the most abundant immune cell subsets in the lung and are among the first cells that are infected with Mtb ^5^. Neutrophils play an important role in bacterial control during acute infection, however, they can release reactive oxygen species (ROS), DNA, myeloperoxidase, cathelicidins and S100 proteins that can result in excessive inflammation and damage to surrounding tissue ^6, 7^. NETosis is the release of nuclear DNA and associated proteins i.e histones, called neutrophil extracellular traps (NETS) from the cell in response to bacterial, viral or stress induced conditions ^8^. The size of the NETS released is dependent on the size of the pathogen ^9^, which is important in combatting infection. However, NETosis has also been shown to contribute to tissue damage in various diseases ^10– 12^. Studying neutrophils in a clinically relevant environment is challenging due to their short life-span and the various complex triggers of activation, including respiratory stress, damage-signalling or prior activation to pathogenic stimuli resulting in ROS associated activities ^13^.

Neutrophilia, which is a characterized by an abundance of neutrophils above what is considered to be a normal count, has been associated with TB and it is therefore important to understand mechanisms underlying neutrophil mediated contribution to disease pathogenesis ^14, 15^. In previous findings, we reported that across cellular, caseous and cavitary granulomas, proteins associated with neutrophils were abundant in necrotic regions ^16^. Other studies have also reported presence of neutrophils around caseous regions ^4^ and in the border of necrotizing tissue ^6^. However, whether neutrophils are directly causing caseation and how it is mediated remains a gap in our knowledge. Our project is therefore aimed at determining expression profiles of neutrophil associated genes in blood from healthy, LTBI and TB participants as potential markers of the disease. We also intend to investigate the role of neutrophils during TB pathogenesis by exploring, *in-vitro*, the dynamics of NETosis, in neutrophils infected with Mtb and to target drivers of the cell death as host directed therapies (HDT).

## Methods

### Study design and setting

This is a prospective study that includes participants recruited from healthcare facilities that service the eThekwini District in KwaZulu-Natal (KZN), South Africa. Participants were recruited from Kwadabeka Clinic and Prince Zulu Communicable Disease Centre in Durban. South Africa has a TB prevalence rate of 737 in 100 000 in 2017 ^17^ with eThekwini described as having the highest TB prevalence rate in KZN. This study was approved by the Biomedical Research Ethics Committee (BREC) at the University of KwaZulu-Natal (BE022/13 and BE00003365/21).

### Study size and ethics

**Figure 1.**
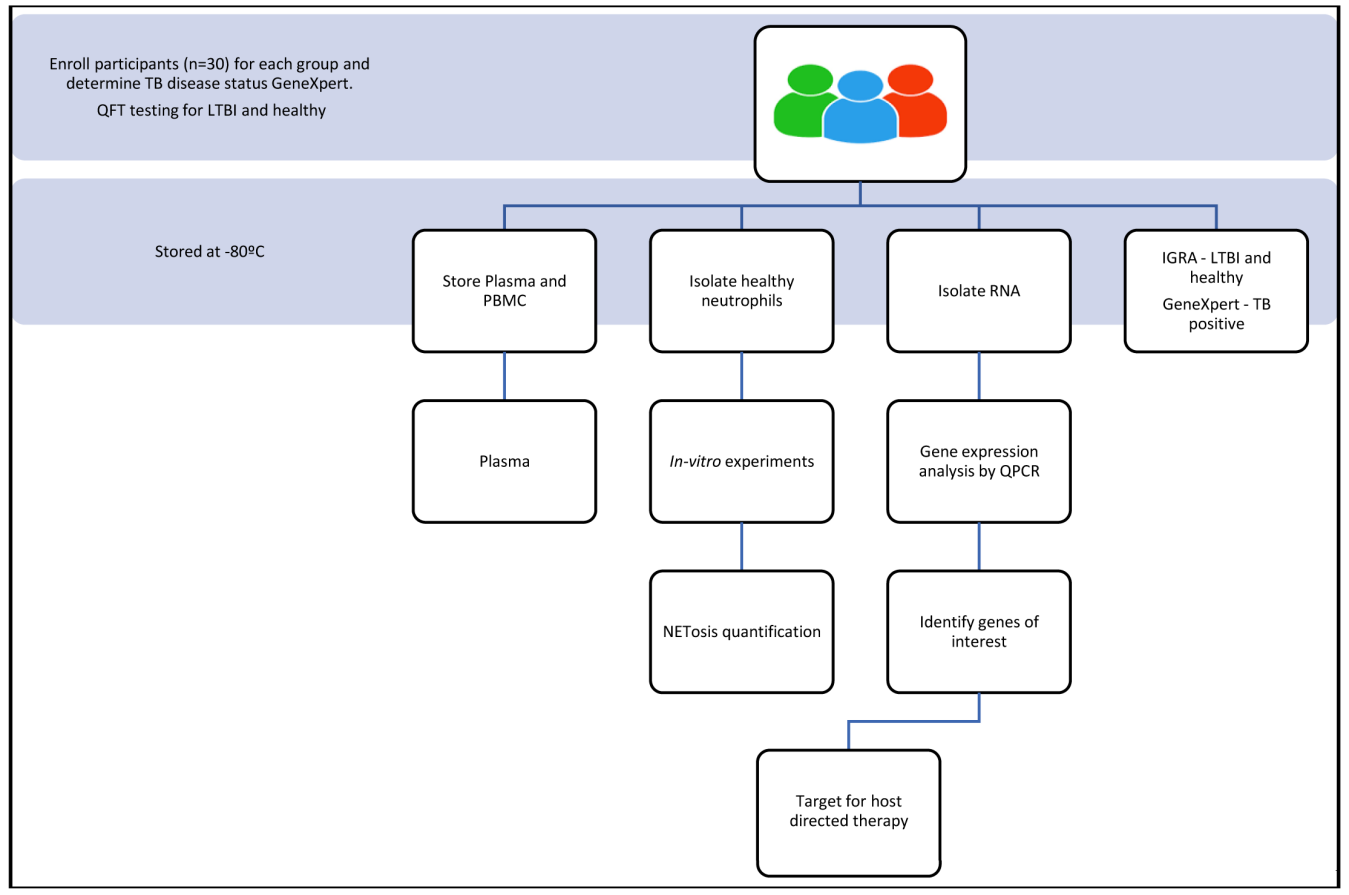
Schematic of the study design and experimental process that will be followed.

**Figure 2.**
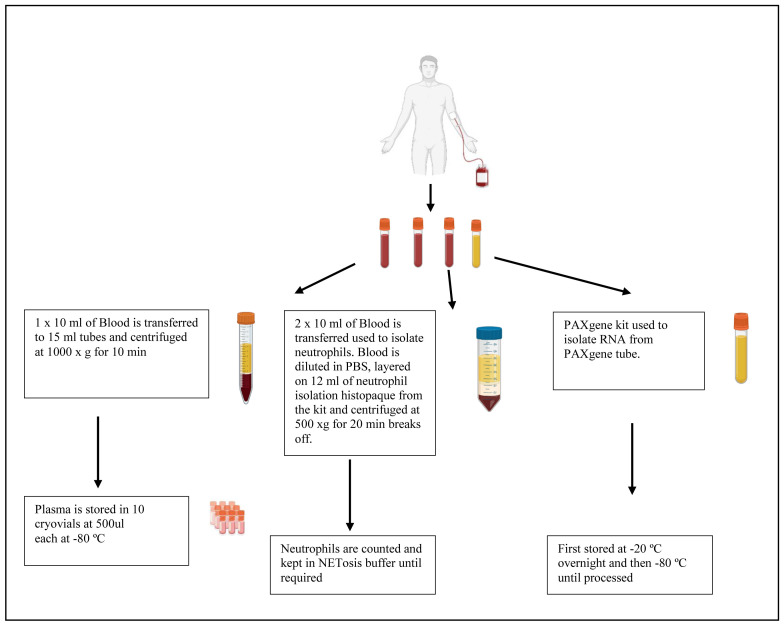
Schematic of plasma, neutrophil and RNA isolation from whole blood obtained from study participants.

A qualified nurse will enrol participants into the study after explaining the informed consent procedure according to good clinical health practices ( Figure 1). Written consent will be obtained from all participants or their legal guardians. Participants will be incentivised for participating in the study according to the ethics that have been approved through the University of KwaZulu-Natal. Participants are informed that their biological samples will be stored for future use, including their genetic information, which will be used for research. A volume of twenty millilitres of blood will be used for neutrophil isolation. Plasma is collected and stored at -80°C ( Figure 2). Approximately thirty participants will be recruited per group.

### Sample processing

Samples that are obtained will be assigned a unique participant identification (PID). A maximum volume of forty millilitres of blood will be drawn from each participant. Two and a half millilitres of blood will be stored for PAXgene processing in order to isolate ribonucleic acid (RNA) for genetic studies. PAXgene tubes are stored at -20°C overnight at angle and transferred to the -80 °C freezer the following day until processing as per the manufacturer’s instructions ( Figure 2). Twenty millilitres of blood will be collected and used to isolate peripheral blood mononuclear cells (PBMCs) or neutrophils and the remaining ten millilitres of blood will be used to collect plasma.

### Neutrophil isolation

A volume of blood ranging between ten and twenty millilitres of blood will be collected in heparin collection tubes ( Figure 2). The blood will be transferred to 50 ml conical tubes. A volume of ten millilitres of 0.01 M phosphate buffered solution (PBS) will be added to the blood collection tubes, to remove any blood remnants and then added to the 50 ml conical tube. A volume of twelve millilitres of the Cell-based Assay Neutrophil Isolation Histopaque (Sigma-Aldrich, Germany) will be placed in a new conical tube at room temperature (RT). The diluted blood will be pipetted on top of the histopaque and centrifuged at 800 x g for 20 minutes at RT, with the centrifuge setting of acceleration slow and deceleration off. The yellow and clear upper layers will be aspirated, leaving only the red blood cell (RBC) layer and neutrophils. A volume of twenty-five millilitres of the RBC lysis buffer (1x solution) will be added to the RBC and neutrophil layer and mixed. The mixture will be incubated at RT for 10 minutes. Following incubation, the cells will be centrifuged at 250 x g for 5 minutes at RT to pellet the neutrophils. The supernatant will be aspirated without disturbing the pellet. The pellet will be resuspended in 25 ml of 0.01 M of PBS and neutrophils counted using a hemocytometer.

### PBMC isolation

To isolate PBMC from the blood, 10 ml of blood will be added to twenty five millilitres of 0.01 M PBS (warmed to RT) (Sigma-Aldrich, USA). The diluted blood will then be layered onto fifteen millilitres of histopaque (warmed to RT) (Sigma-Aldrich, USA). A density gradient will be formed which will allow the cells to separate. The layered cells will be centrifuged at 800 x g for 30 minutes with the centrifuge setting of deceleration offand slow acceleration. Following centrifugation, the whitish layer (buffy layer) will be aspirated to a new 50 ml tube and topped up with PBS and centrifuged at 300 x g for 10 min, with acceleration and deceleration at maximum. The supernatant will be discarded, and the cells will be resuspended in PBS before being counted. The cells will be centrifuged at 300 x g for 10 min with maximum acceleration and deceleration. Freeze media will be prepared by adding 500µl of dimethyl sulfoxide (DMSO) (Sigma-Aldrich, USA) to four and half millilitres of fetal calf serum (FCS) (Sigma-Aldrich, USA). The supernatant will be removed, and the cells will be stored in 1 ml of freeze media at a cell concentration of 10 million cells per ml in a cryovial labelled with the PID and date and stored in liquid nitrogen. Plasma will be isolated by transferring ten millilitres of blood to a tube, which will be centrifuged at 1000 x g for 10 minutes, with maximum acceleration and with deceleration off. Ten cryovials labelled with the PID and date, are used to store plasma at -80°C at a volume of 500µl.

### RNA extraction

All surfaces and pipettes will be decontaminated with RNAse away before RNA isolation will be done. RNA will be extracted using the PAXgene kit and the corresponding protocol. Briefly, blood from TB, LTBI and healthy participants will be collected in PAXgene tubes that contain anticoagulant to ensure the stability of the ribonucleic acid (RNA). The blood will be stored at 18-25°C during transit and upon arrival will be stored at -20°C at an angle on a wired rack for 24 hours before being transferred to the -80°C freezer. The frozen blood in the PAXgene tubes will be thawed in a water bath at 22–23°C for 20 minutes and the PAXgene tube will be inverted 8–10 times to before RNA isolation proceeded. Once thawing and inverting of the tube is complete, the PAXgene tube is then left at room temperature (RT) for a further 2 hours to ensure complete cell lysis. RNA will be isolated from the blood using the PAXgene kit according to the manufacturer’s instructions (PreAnalytiX, Hombrechtikon, Switzerland).

### RNA quality

To determine the RNA quality, we will be using a Nanodrop Lite Spectrophotometer (Thermofisher ™, Switzerland) and the absorbance will be read at 260nm. The sample will be diluted (dilution factor 2) with 10mM of Tris-hydrochloride, pH 7.5. The relationship between the absorbance and the RNA yield will be taken as A260 of 1 is equal to 44ug/ml, according to the manufacturer’s instructions.

### cDNA synthesis

The iScript cDNA synthesis kit will be used for cDNA synthesis as per manufacturer’s instructions (Biorad, California, USA). The 5 x iScript reaction mix will be left to thaw at room temperature. The reaction will be set-up in sterile PCR tubes as follows; 500 ng of RNA will be added to each tube, followed by 4 µl of 5x iScript reaction mix, 20 µl of RNase free water and 1 µl of iScript reverse transcriptase. The total reaction mix will be 20 µl per tube. The tubes will be placed in a PCR machine and run on the thermocycler (Biorad, California, USA) using the following runs, 5 min at 25°C, 30 min at 42°C, 5 min at 85°C and on hold at 4°C. The resultant cDNA will be diluted 10x by adding 1 µl of cDNA to 9 µl of nuclease free water.

### RT-QPCR for gene expression

Qualitative polymerase chain reaction (qPCR) will be done on candidate genes as per the manufacturer’s instruction to determine the expression of the candidate genes in TB, LTBI and healthy participants. Briefly, half a microlitre of reverse primer, half a microlitre of the forward primer, three microlitres of nuclease free water and five microlitres of iTaq Universal SYBR green supermix (Biorad, California, USA) will be added to each well. A volume of nine microlitres of the mastermix will be added to each well. One microlitre of the diluted sample will be added to each well. The plate will be run on the Biorad CFX 96 thermocycler (Biorad, California, USA) according to the following protocol, 30 sec at 95°C, 5 sec at 95°C, 30 sec at 59°C, for 39 cycles. The melt curve analysis will be done at 65–95°C at 0.5°C increments.

### NETosis quantification

**Figure 3.**
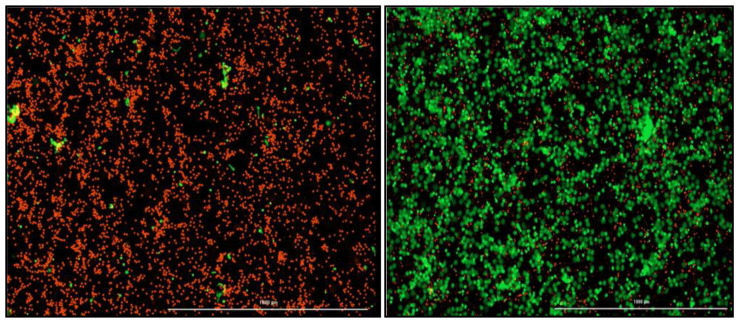
Images acquired with the Biotek Cytation 5 (1000µM) to visualize and quantify NETosis. Red indicates intact neutrophils. Green indicates neutrophils undergoing NETosis and extruding their cellular DNA. (Left) Negative control which is only healthy neutrophils with no stimulation. (Right) Positive control, PMA stimulated neutrophils.

A NETosis imaging kit (Cayman Chemical, Michigan, USA) will be used to quantify the amount of NETosis occurring in TB patients (n=4) compared to healthy participants (n=4). The assay will be performed as per manufacturer’s instructions. Briefly, 6 × 10 ^6^ neutrophils isolated on the same day, will be transferred to a clean tube and adjusted in 0.01M PBS to 1 × 10 ^6^ cells/ml. a volume of six microlitres of Permeable Nuclear Red Reagent (5mM) will be added to each well and incubated in the dark at 37°C for 15–30 minutes. A volume of 25ml of PBS will be added to the cells, centrifuged at 250 x g and the supernatant aspirated. The cells will be resuspended in twelve microlitres (0.5 × 10 ^6 ^cells/ml) of NETosis Imaging buffer (1x). a volume of twelve microlitres of Extracellular Nuclear Green Reagent (5mM) will be added to the tube and mixed before the cells are seeded into the wells at 100 µl/well in a flat-bottom 96 well plate (Greiner Costar, Sigma-Aldrich, Germany). A volume of 100 µl of stimuli or NETosis Imaging Buffer (1x) will be added to each well for a final volume of 200 µl. The plate will be centrifuged at 250 x g to pellet the cells at the bottom of the plate. The BioTek cytation 5 (Biotek, Vermont, USA) which uses brightfield and fluorescence microscopy will be used to image activity every 30 min–1 hour for six to twelve hours at a temperature of 37°C and 5% CO _2_. Green fluorescence indicates NETosis activity and red fluorescence is a marker for the nucleus ( Figure 3). The neutrophils will be treated with optimized concentrations of various NETosis inhibitors depending on the chosen gene targets.

### Data analysis

Student t-tests will be used to analyse the difference in gene expression between healthy, LTBI and TB participants. The data will be reported with the median and interquartile range. Pearson R Correlation or a Spearman R Correlation analysis will be done to determine if there is an association between protein and gene expression of specific NETosis associated protein and genes. All data will be analysed using Graphpad Prism v 9 software. 

## Discussion

Proteomic analysis of lung tissue laser microdissections of various granulomas showed that proteins associated with neutrophils were abundant in necrotic regions of caseous and cavitary granulomas ^16^. This indicated that neutrophils potentially contribute to necrotic damage observed in cavitary granulomas, due to their presence in these regions. This provides a potential link of the neutrophils with increased TB pathogenesis, hence the need to investigate their role during TB as potential target for therapy or as biomarkers. Identifying factors or mechanisms that contribute to lung tissue damage is vital in identifying markers of disease progression.

Neutrophils have been identified in the regions that border necrotizing tissue in other diseases ^18^ and how they contribute to caseation during TB infection remains poorly understood. Recent studies have also identified the role of DNA-MPO ^12, 19^ or histones complexes ^11, 20^, ROS pathways ^10^ and neutrophil elastase ^21^ in tissue damage associated with various respiratory infections and as markers of disease progression. Studies in C3HeB/FeJ mice infected with Mtb have also shown that neutrophils contribute to necrosis and liquefaction of granulomas mediated by ROS events ^22^. These studies suggest that similar mechanisms mediated by neutrophil derived proteins and cell death activities may be driving TB lung pathology in humans.

NETosis proteins have been detected in the serum of individuals with acute respiratory distress syndrome associated pathology ^23^. However, whether NETosis directly contributes to damaging pathology observed in TB granulomas is not well known. To validate this, our initial aim is to establish whether NETosis associated genes are present in the blood and are differentially expressed in LTBI, TB and healthy participants. In addition, we aim to determine whether expression of specific genes associated with NETosis correlates with the spectrum of disease observed during TB infection (as reviewed by 24, 25. In addition, these unique gene expression profiles may be used to identify potential biomarkers of disease progression that may be used to diagnose TB at point of care.

Ongoing clinical trials are investigating the use of NETosis specific gene inhibitors to reduce tissue damage associated with NETotic events ^26– 30^. Therefore, identifying potential targets for HDTs that may serve as adjunct therapies to current antibiotic regimens remains vital in combating the disease.

Due to the heavy TB disease burden in KZN, the study participants that will be enrolled in this study will provide important and relevant information regarding the tissue destruction mechanism that contributes to disease progression. A limitation of this study is that we will not be able to obtain matched blood samples from lung tissue donors. Another limitation of this study is that when performing the NETosis experiments it is difficult to distinguish between LTBI volunteers and those who are healthy in a timeous manner. This limits the ability of us to obtain a healthy blood volunteer to have an appropriate control.

The aim of this study is to identify potential biomarkers of TB disease progression that can lead to better diagnosis and ultimately, treatment of TB. We hope to characterize drivers of neutrophil mediated damage by developing a neutrophil *in-vitro* infection model. These findings may contribute to a better understanding of the neutrophil driven mechanisms that lead to damage often observed in the lungs of individuals with TB disease.

## Study status

Recruitment of the study participants has been completed. The component of the study aimed to determine neutrophil related genes in healthy, LTBI and TB patients has been completed. The in vitro model of neutrophil Mtb infection has been optimized and detailed experiments on kinetics and mechanisms of TB induced of NETosis are ongoing.

## Data availability

Data will be stored and available through the Africa Health Research Institute according to the Institute’s protocols which ensure participant privacy and rights protection. Data generated will also be deposited into relevant public data repositories.
