# Supplementary material for: Investigating neutrophil cell death in TB pathogenesis
Source: Gates Open Res. 2022 Apr 29;5:175. Originally published 2021 Dec 7. [Version 2] doi: 10.12688/gatesopenres.13472.2 (PMC8990965; doi:10.12688/gatesopenres.13472.2)
Supplement: Peer review history

# Peer review: Reviewer response for version 2

**DOI:** 10.21956/gatesopenres.14919.r31993

**Published:** 2022-05-09

**Authors:** Ashwani Kumar

**Affiliations:** 1 Council of Scientific and Industrial Research, Institute of Microbial Technology, Chandigarh, Chandigarh, Punjab and Haryana, India

My suggestions have been incorporated into the revised study protocol.

Is the study design appropriate for the research question?

Yes

Is the rationale for, and objectives of, the study clearly described?

Yes

Are sufficient details of the methods provided to allow replication by others?

Yes

Are the datasets clearly presented in a useable and accessible format?

Yes

Reviewer Expertise:

TB Pathogenesis

I confirm that I have read this submission and believe that I have an appropriate level of expertise to confirm that it is of an acceptable scientific standard.

# Peer review: Reviewer response for version 1

**DOI:** 10.21956/gatesopenres.14730.r31865

**Published:** 2022-04-07

**Authors:** Delia Goletti

**Affiliations:** 1 Translational Research Unit, National Institute for Infectious Diseases (INMI), Rome, Italy

This study is on a project that is going to be done on the evaluations of genes associated with neutrophil mediated cell death. The hypothesis is that their study might be useful as potential biomarkers of pathological damage and disease progression, as well as targets for host-directed therapy.

 The hypothesis and protocol of the study are well written. 

**Few comments**

**Methods:**

- Please comment on how the number of patients and controls was chosen.
- Please comment on the definition of patients with latent tuberculosis infection, and which tests/radiological tools you will use to define them
- Please comment on the definition of patients' active tuberculosis, and which tests/radiological tools you will use to define them

Is the study design appropriate for the research question?

Yes

Is the rationale for, and objectives of, the study clearly described?

Yes

Are sufficient details of the methods provided to allow replication by others?

Yes

Are the datasets clearly presented in a useable and accessible format?

Yes

Reviewer Expertise:

Immunology of tuberculosis and COVID-19 and Echinococcosis.

I confirm that I have read this submission and believe that I have an appropriate level of expertise to confirm that it is of an acceptable scientific standard.

# Response

**Published:** 2022-04-25

**Authors:** Mohlopheni Jackson Marakalala

**Affiliations:** Africa Health Research Institute, South Africa

**Reviewer 2**

  

- **Please comment on how the number of patients and controls was chosen.**
- **Please comment on the definition of patients with latent tuberculosis infection, and which tests/radiological tools you will use to define them**
- **Please comment on the definition of patients' active tuberculosis, and which tests/radiological tools you will use to define them**

Response to reviewer 2:

 We appreciate the useful comments by Reviewer 2 which we believe will help strengthen our manuscript.

 We will not be using a radiological test to confirm TB infection but rather GeneXpert. Those individuals who are GeneXpert positive will be recruited to the TB group. Those individuals who will be recruited to the healthy group were further tested to determine whether they were perhaps latently infected with TB using the quantiferon (QFT) test. Those that have a positive QFT result will be recruited to the LTBI group (n=30) and those that have a negative QFT result will be recruited to the healthy group (n=30).

 Please see below edits to the manuscript on lines 86-91:

 “ *Individuals who are newly diagnosed with active TB, determined as GeneXpert positive and who have not received treatment, will be recruited to the TB group of the study (n=30). Individuals who are healthy will be recruited to the healthy group as determined by their quantiferon (QFT) results. If the QFT result is positive those individuals will be recruited to the LTBI group (n=30) and those who are QFT negative will be recruited to the healthy group (n=30)*.”

 The size of the group was determined based on our discovery proteomics data. We decided to investigate the neutrophil specific protein responses in a small cohort that would allow us to determine if similar responses were seen in local populations within KZN. We acknowledge that the sample size is small and is a limitation of this study. However, the findings of this study could be validated in a larger cohort, should promising biomarkers of disease progression be discovered.

# Peer review: Reviewer response for version 1

**DOI:** 10.21956/gatesopenres.14730.r31616

**Published:** 2022-02-02

**Authors:** Ashwani Kumar

**Affiliations:** 1 Council of Scientific and Industrial Research, Institute of Microbial Technology, Chandigarh, Chandigarh, Punjab and Haryana, India

Study protocol titled “Investigating neutrophil cell death in TB pathogenesis” provides details of methods related to the assessment of differential gene expression in PBMCs and their association with latency of TB (if any). It also provides details of methods that could assist in deciphering genes involved in NETosis. Overall the manuscript is well written and provides sufficient details of methods. I would like to suggest a few minor changes to the manuscript.

1. Study size and ethics sub-headings of the Methods section shall provide sample size, expected numbers of individuals that will be enrolled in the study and its justification.
2. Background section, line 1, authors could use a recent TB report 2021 instead of Global TB report 2020.
3. There are a few grammatical errors in the methods sections, correcting these may improve the readability of the manuscript. As an example in the “study size and ethics” sub-heading,  “plasma is collected’ is used. It could be changed to “plasma will be collected”.
4. Authors should use the convention of a space between numbers and units eg., “5 mM” instead of  “5mM”.

Is the study design appropriate for the research question?

Yes

Is the rationale for, and objectives of, the study clearly described?

Yes

Are sufficient details of the methods provided to allow replication by others?

Yes

Are the datasets clearly presented in a useable and accessible format?

Yes

Reviewer Expertise:

TB Pathogenesis

I confirm that I have read this submission and believe that I have an appropriate level of expertise to confirm that it is of an acceptable scientific standard.

# Response

**Published:** 2022-04-25

**Authors:** Mohlopheni Jackson Marakalala

**Affiliations:** Africa Health Research Institute, South Africa

We appreciate the useful comments by Reviewer 1 which we believe will help strengthen our manuscripts.

 Reviewer 1

**1. Study size and ethics sub-headings of the Methods section shall provide sample size, expected numbers of individuals that will be enrolled in the study and its justification.**

 Response to question 1:

 Please see below edits to the manuscript on line 86-91:

 “Individuals who are newly diagnosed with active TB and are GeneXpert positive, will be recruited to the TB group of the study (n=30). Individuals who are healthy will be recruited to the healthy group as determined by their quantiferon (QFT) results. If the QFT result is positive those individuals will be recruited to the LTBI group (n=20) and those who are QFT negative will be recruited to the healthy group (n=20).”

**2. Background section, line 1, authors could use a recent TB report 2021 instead of Global TB report 2020.**

 A more recent TB report has been used. Please see line 39 and 380.

**3. There are a few grammatical errors in the methods sections, correcting these may improve the readability of the manuscript. As an example in the “study size and ethics” sub-heading,  “plasma is collected’ is used. It could be changed to “plasma will be collected”.**

 The manuscript has been edited to change the tense to the correct grammatical tense.

**4. Authors should use the convention of a space between numbers and units eg., “5 mM” instead of  “5mM”**

 The manuscript has been edited to include a space between the number and units of measurements.

 ​​​​​​
